# Transcranial Direct Current Stimulation over the Posterior Parietal Cortex Increases Nontarget Retrieval during Visual Working Memory

**DOI:** 10.1523/ENEURO.0265-24.2024

**Published:** 2024-11-14

**Authors:** Shengfeng Ye, Menglin Wu, Congyun Yao, Gui Xue, Ying Cai

**Affiliations:** ^1^Department of Psychology and Behavioral Sciences, Zhejiang University, Hangzhou 310000, China; ^2^State Key Laboratory of Cognitive Neuroscience and Learning and IDG/McGovern Institute of Brain Research, Beijing Normal University, Beijing 100875, China

**Keywords:** content–context binding, occipital cortex, posterior parietal cortex, transcranial direct current stimulation, visual working memory

## Abstract

Visual working memory (VWM) requires precise feature binding. Previous studies have revealed a close relationship between the posterior parietal cortex (PPC) and feature binding during VWM; this study further examined their causal relationship through three transcranial direct current stimulation (tDCS) experiments. In Experiment 1 (*N* = 57), participants underwent three sessions of tDCS separately, including PPC stimulation, occipital cortex stimulation, and sham stimulation, and completed delayed estimation tasks for orientations before and after stimulation. Results showed that tDCS over PPC selectively prolonged recall response time (RT) and increased the probability of nontarget responses (a.k.a. failure of feature binding, *p*NT). In Experiment 2 (*N* = 29), combining metacognition estimation, we further investigated whether the effects of PPC stimulation were attributed to misbinding (i.e., participants self-reported “remembered” in nontarget responses) or informed guessing trials (participants self-reported “forgotten” in nontarget responses). We replicated the main findings in Experiment 1 and observed greater tDCS effects of PPC on RT in informed guessing trials while there are comparable effects on *p*NT in these two types of trials. In Experiment 3 (*N* = 28), we then examined whether the tDCS effects over PPC specifically influenced the memory retrieval process by using a change detection task. We found that PPC stimulation did not influence the recognition RT or accuracy. Together, this study provided direct causal evidence supporting the specific involvement of PPC in feature binding during VWM retrieval, from both aspects of speed and response preference, expanding our understanding of the neural basis of feature binding in VWM.

## Significance Statement

Visual working memory (VWM) enables humans to temporarily store and process visual information, which requires accurate binding of items to their unique context. Accumulating studies posited that the posterior parietal cortex (PPC) is closely related to this binding process, the current study further examined their causal relationship. Through three strictly within-subject well–designed noninvasive neural stimulation experiments, we found that PPC stimulation selectively increased response time and binding error during VWM. Moreover, we found these changes were modulated by individual metacognition and only occurred during memory recall instead of recognition. Together, our results provided strong evidence that PPC is causally involved in the binding process during VWM retrieval.

## Introduction

Visual working memory (VWM), a process of storing and processing visual information temporarily, is an essential basis of higher-level cognitive processes (Baddeley and Hitch, 1974). Precise content–context binding is critical for multi-item VWM. An interference model indicates that VWM capacity is mainly limited by the binding interference between the content of multiple items and their context information (Oberauer and Lin, 2017). Moreover, impaired binding ability is a typical symptom in varying neurodegenerative disorders (Mayer et al., 2012; Kirova et al., 2015). Thus, understanding the mechanism underlying content–context binding in VWM is always an important theoretical question.

Increasing studies have demonstrated the relationship between posterior parietal cortex (PPC) activity and feature binding during VWM. In early studies, patients with right PPC lesions exhibited a decreased accuracy in tasks requiring multi-item bindings (Ashbridge et al., 1999) and reported illusory feature conjunctions during recall (Braet and Humphreys, 2009).Although earlier functional magnetic resonance imaging (fMRI) and electroencephalograph (EEG) studies have revealed that PPC activity was tightly correlated with the number of items kept during VWM (Todd and Marois, 2004), recent fMRI studies showed that holding the memorized items constant, PPC activity was higher when binding demands increased, suggesting that PPC played a closer role in the binding process (Gosseries et al., 2018; Cai et al., 2020).

Besides, modeling studies have advanced the relationship between PPC and feature binding to the individual and trial level. Bays et al. (2009) proposed a multiple-component mixture model to estimate individuals’ probability of nontarget responses, which reflected the failure of binding in a delayed estimation recall task. Accordingly, Cai et al. (2020) found that the neural decoding strength of the context information (i.e., location) in PPC predicted nontarget response rates. Moreover, Schneegans and Bays (2016) proposed a novel model to estimate the probability of nontarget recalls at the trial level. Combining this model and metacognitive reports, researchers further identified two types of nontarget responses that reflect different cognitive processes (Pratte, 2019). In the misbinding condition, individuals are unconscious of recalling a nontarget item and report a high recall confidence (i.e., “remember trials”). In the informed guessing condition, in contrast, they know they may wrongly report a nontarget item and thus report low recall confidence (i.e., “forgotten trials”). Recently, Mallett et al. (2022) observed that participants responded with high confidence in about three-quarters of nontarget trials in a delayed estimation task. More importantly, they found that nontarget items could be decoded in PPC since early maintenance. Unfortunately, this study did not compare the differences between these two types of nontarget responses; thus the involvement of PPC in different nontarget responses was still unclear.

Transcranial direct current stimulation (tDCS) is a noninvasive neuromodulation technique to explore the causal relationship between neural activities and cognitive processes. Typically, tDCS involves a two-electrode setup on the scalp to deliver weak direct current, with the anodal stimulation increasing cortical excitability while the cathodal stimulation inhibiting the excitability (Nitsche and Paulus, 2000; Stagg and Nitsche, 2011). Currently, tDCS research testing the relationship between PPC and VWM has focused on memory capacity, while the results were quite controversial (Li et al., 2017; Wang et al., 2019; Dumont et al., 2021; Jiang et al., 2023). For these inconsistencies, researchers have proposed some important individual differences modulating tDCS effects, such as the VWM baseline (Tseng et al., 2012; Hsu et al., 2014), remember-subset strategy (Wang et al., 2020), and biorhythms (i.e., morning vs afternoon; Salehinejad et al., 2019; 2023). In sum, few tDCS studies have examined the causal relationship between PPC and feature binding, and to answer this question, it is critical to control irrelevant variables and consider potential individual differences that affected tDCS effects.

In this study, we systematically investigated the causal relationship between PPC and feature binding in VWM through three tDCS experiments. In Experiment 1, combined with the three-factor mixture model, we examined the effect of tDCS over PPC on binding process in a delayed estimation task and explored whether individual differences in capacity and recall strategies affected tDCS effect. In Experiment 2, we tried to replicate the results of Experiment 1 and further investigated the involvement of PPC in two types of nontarget responses (i.e., misbinding and informed guessing), by integrating remembered-forgotten self-reports. In Experiment 3, we examined whether the tDCS effects specifically during memory retrieval by using a change detection task (e.g., recognition) instead of a delayed estimation task (e.g., recall). If tDCS changed VSTM maintenance, similar effects should be observed in both tasks; otherwise, no such effect would be detected in the recognition task.

## Materials and Methods

### Experiment 1

#### Participants

Fifty-eight university students participated in Experiment 1 [34 females; mean (M), 20.10 years; SD, 1.40]. All participants were right-handed, had normal or corrected-to-normal vision, and reported no history of neurological or psychiatric disorders. Before the experiment, participants signed a written informed consent required by the institutional review board of the Department of Psychology and Behavioral Sciences, Zhejiang University. Participants received monetary compensation for participation (¥30 per hour).

#### Experimental procedure

We modified a delayed estimation task for orientations from previous studies to estimate the tDCS effects on the feature binding process in VWM, and only high memory loads were included given researchers have reported higher chances of tDCS effects in the super-capacity condition (Wang et al., 2019, 2020). In each trial, after a 500 ms white central fixation (0.75 × 0.15°), an array of six or eight white, randomly oriented bars (2 × 0.3°) were presented for 200 ms. All bars were uniformly presented on an invisible circle (centered on the screen, radius of 6°), and orientations of these stimuli were randomly selected from 10 to 170° with at least 10° apart. After a 1 s delay, participants were instructed to recall the orientation of the bars at the probed position as precisely and fast as possible, by moving the mouse and clicking the left bottom on the white circular probe (radius of 2°). The maximum response time (RT) was 8 s. Participants completed the task before and after each tDCS session for ∼20 min. Each session consisted of 240 trials which were divided into four blocks, with two memory loads randomly mixed. Participants completed 24 practice trials in each memory load condition before the formal experiment to become familiar with the task.

Experimental stimuli were displayed on a 17 in color screen running MATLAB 2019a (MathWorks) and the Psychophysics Toolbox 3.0.12 (Brainard, 1997). Participants were seated 60 cm away from the monitor (resolution, 1,920 × 1,080; refresh rate, 60 Hz) in a quiet room and were instructed to keep their eyes fixed on the center of the screen throughout the experiment. At the end of the experiment, participants completed a five-point scale to evaluate how often they use remember-all strategy or remember-subset strategy under different memory loads (1, always “remember-all”; 5, always “remember-subset”).

#### tDCS setup

**Figure 1.**
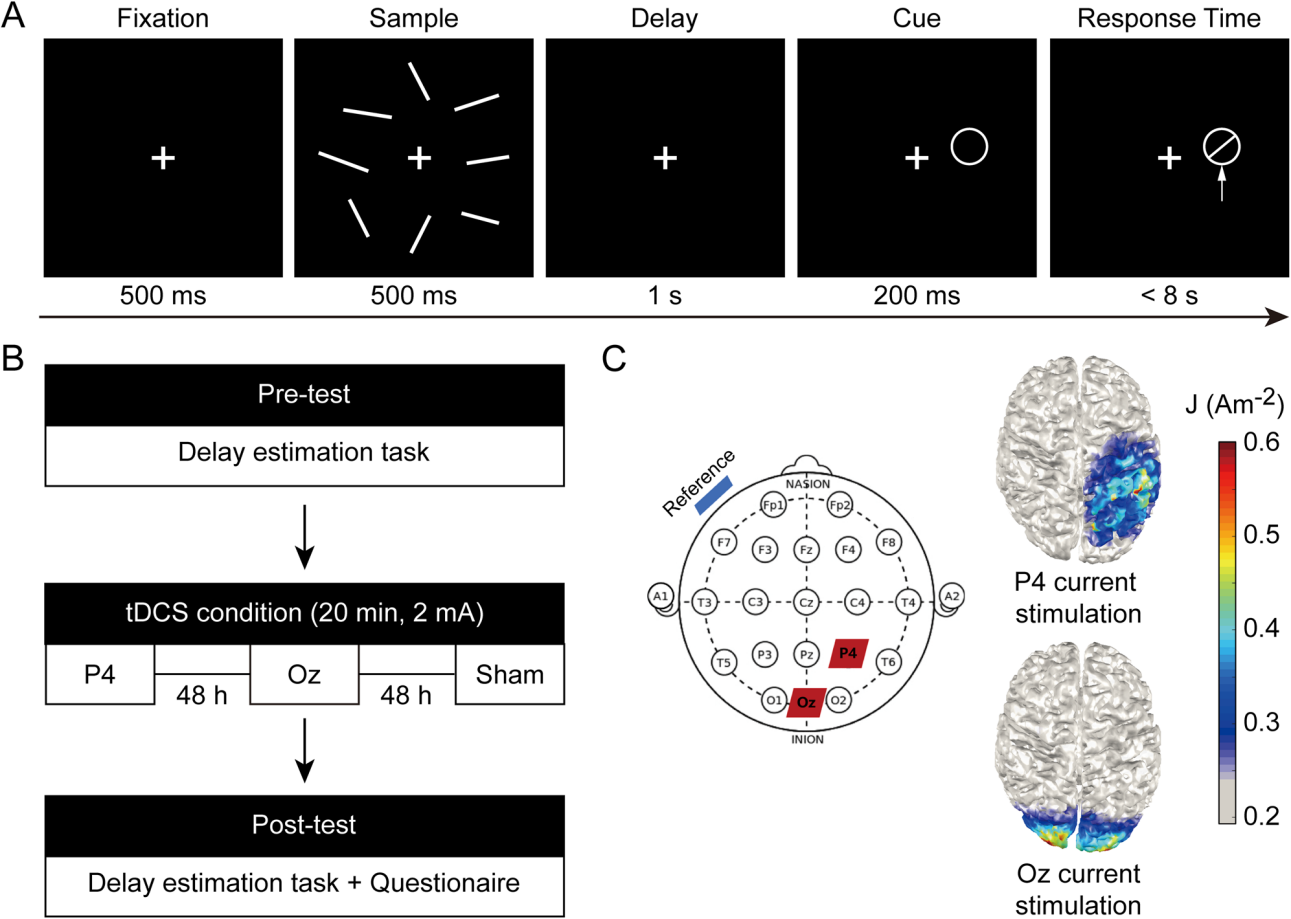
***A***, Schematic diagrams of the delay estimation task. ***B***, tDCS procedures. ***C***, The placement of tDCS electrodes (left) with red patches representing the anodal electrode (P4 and Oz) and a blue patch representing the reference electrode (left cheek) and cortical current density distributions from an overhead view (right).

Participants underwent three tDCS sessions, including PPC stimulation, occipital cortex (OCC) stimulation, and sham stimulation. The order of stimulations was counterbalanced across participants, and three stimulations were performed separately with an exactly 48 h interval to minimize potential carryover effects and influences of circadian rhythms (Fig. 1*B*). tDCS was delivered by the DC-STIMULATOR MC device (neuroConn) using a pair of plastic electrodes (5 × 7 cm^2^) in a saline-soaked synthetic sponge. In PPC stimulation, the anodal electrode was placed over P4 according to the international 10–20 EEG electrode system (Hsu et al., 2014; Li et al., 2017; Wang et al., 2019, 2020), the reference electrode was placed over the left cheek. Then a 20 min, 2 mA current was applied, with a linear fade in and fade out of 30 s which could minimize the uncomfortable feelings of sudden current changes. The choice of stimulation parameters referred to previous studies and guaranteed long-lasting stimulation effects during the poststimulation task (Wang et al., 2019, 2020). In OCC stimulation, the only difference is the anodal electrode was placed over Oz (Makovski and Lavidor, 2014; Li et al., 2017). For the sham condition, the anodal electrode was randomly placed over P4 or Oz, and the stimulation was only delivered within the first and last 30 s to simulate the itching feelings during active stimulation. During all stimulations, participants sat and took a rest. The current density distributions for tDCS settings were presented using COMETS, an open-source toolbox based on MATLAB (http://www.cometstool.com; Lee et al., 2017; Fig. 1*C*).

### Data analysis

#### Estimations of behavioral performance

We calculated the RT and the recall error under different memory loads. RT was defined as the duration between the cue onset and response confirmation, and the recall error was obtained as the angular distance between the reported orientation and the targeted orientation. Then, we adopted the three-factor mixture model (Bays et al., 2009) and fit the distribution of raw errors to obtain the precision of target responses (κ), the probability of target responses (*p*T), the probability of nontarget responses (*p*NT), and the probability of guess responses (*p*U). Among these measures, a higher *p*NT indicated a higher probability of content–context binding errors. Memory capacity was calculated as *p*T × memory load according to previous research (Zhang and Luck, 2008). One participant was excluded due to poor working memory performance (capacity <1). Then, 57 participants were included for further analysis.

#### Estimations of tDCS effects

To examine tDCS effects, we first compared the behavioral parameters between active stimulation (PPC or OCC stimulation) and sham stimulation separately. For each behavioral parameter, we conducted a three-way repeated–measure analysis of variance (ANOVA) of stimulation condition (active vs sham), test time (pretest vs posttest), and memory load (Set Size 6 vs Set Size 8). If interaction effects were significant, we then performed two-way repeated–measure ANOVAs investigating the tDCS effects under each load. Furthermore, if any tDCS effect was significant in active stimulation, we tested whether this effect was region-specific by comparing tDCS effects over PPC and OCC with repeated–measure ANOVAs as well as calculating Pearson’s correlation between PPC and OCC tDCS effects (i.e., the behavioral changes before and after stimulation). We reported *p* values, effect size, and Bayesian factor (BF) for all statistical analyses.

#### Examination of influence factors of tDCS effects

For significant tDCS effects at the group level, we examined the potential influences of individual working memory capacity and recall strategy scores on tDCS effects via Pearson's correlation analyses. Because the capacity measures (i.e., *p*T × each memory load) were highly correlated in three prestimulation sessions and two memory loads (*r*s > 0.379; *p*s < 0.01; BF_10s_ > 10.263), we averaged them as the individual capacity performance. The recall strategy scores were obtained in each memory load, and as expected, there was a higher chance of using the remember-subset strategy in higher memory load conditions [SS6, M (SD), 4.158 (0.841); SS8, M (SD), 3.368 (1.063); difference, *t*_(56)_ = 6.926; *p* < 0.001; BF > 1,000]. Similarly, the recall strategy scores across set sizes were highly correlated (*r* = 0.613; *p* < 0.001; BF_10_ > 1,000), we then averaged them as the individual memory strategy if the tDCS effects were significant in both set sizes, while only using the recall strategy score in the specific memory load (i.e., Set Size 8) if the tDCS effect was load-specific.

### Experiment 2

#### Participants

The sample size was determined using G*Power 3.1 (Faul et al., 2007) based on the effect size of increased *p*NT after PPC stimulation in Experiment 1 (*η*_p_^2^ = 0.119). A minimum sample of 28 participants was required to achieve a power of 90%, with a significance level of 0.05. We recruited 33 healthy university students (14 females; M, 23.10 years; SD, 2.70), and four participants were excluded due to poor task performance, resulting in a final sample of 29 in this experiment. Recruitment and payment criteria were consistent with Experiment 1.

#### Experimental procedure and tDCS setup

The task procedure was similar to Experiment 1, except that participants were required to make metacognitive evaluations of their recall during the task. This design could help us to distinguish misbinding errors and informed guessing (Pratte, 2019). More specifically, in each trial, after 1 s delay, a white circle cueing the location of the probed orientation appeared for 200 ms, and participants were asked to report whether they remembered or forgot the probed orientation by pressing the left or right button of the mouse. The left–right buttons were counterbalanced across participants. Subsequently, participants were instructed to recall the targeted orientation at the probed position within 4 s by moving the mouse and making a confirming click. Finally, participants rated their confidence in a seven-point scale (1, “lowest confidence”; 7, “highest confidence”), which was introduced as a validation of the remember–forget binary forced choice (Fig. 5*A*).

Participants completed six blocks of the delay estimation task before and after stimulation, each block included 60 trials, and each task lasted for ∼20 min. In this experiment, we only focused on the high set size condition (i.e., Set Size 8) and included two tDCS sessions (i.e., PPC and sham). All stimulation setups were similar to Experiment 1 (Fig. 5*B*).

### Data analysis

#### Replicate the overall tDCS effects on RT and nontarget response

Across all trials, we first examined tDCS effects over PPC on prolonged RT and increased *p*NT observed in Experiment 1 by conducting a two-way repeated–measure ANOVA for stimulation condition (PPC vs sham) and test time (pretest vs posttest). For significant two-way interaction effects, we further conducted simple effect tests. Similar to Experiment 1, for significant tDCS effects, we explored the potential influences of capacity and strategy through correlation analysis. Here, we used an advanced model fitting method to obtain all three parameters (*p*NT, *p*T, *p*U) for each trial (Schneegans and Bays, 2016).

#### Compare tDCS effects between remembered and forgotten trials

Trials were divided into remembered trials (trial number, M, 220.974; SD, 12.872) and forgotten trials (M, 131.371; SD, 10.570) based on the binary forced choice. Paired *t* tests revealed that errors for remembered trials were significantly lower than those for forgotten trials [M (SD), 16.280 (5.743) versus 29.208 (7.630); *t*_(28)_ = −16.174; *p* < 0.001; Cohen's *d* = −3.003; BF_10_ > 1,000], and confidence ratings were also significantly higher for remembered trials than for forgotten trials [M (SD), 4.922 (0.960) versus 2.302(0.629); *t*_(28)_ = 15.638; *p* < 0.001; Cohen's *d* = 2.904; BF_10_ > 1,000], suggesting that our metacognition categorization was validated, and forced choice self-reports reflected objective memory. Then, behavioral parameters for each trial type were averaged for follow-up tDCS effect comparisons.

For the tDCS effects on RT and *p*NT, we examined whether these effects differed across trial types through a three-way repeated–measure ANOVA of trial type (remembered vs forgotten), stimulation condition (PPC vs sham), and test time (pretest vs posttest). We further conducted two-way repeated–measure ANOVAs and simple effect tests for stimulation condition and test time in each trial type if the three-way interaction effects were significant. Other parameters were also tested in a similar way.

### Experiment 3

#### Participants

Thirty-four healthy university students (18 females; M, 22.00 years; SD, 2.06) were recruited. Six participants were excluded due to poor performance, leaving 28 participants for the following analysis. The sample size choice, participant recruitment, and payment criteria were consistent with Experiment 2.

#### Experimental procedure

The change detection task for orientations was adapted from a previous study (Gong and Li, 2014; Fig. 6*A*). By including a lure-trial condition (Luck et al., 2009), we can examine the tDCS effects on the feature binding in VWM recognition. The encoding and maintenance periods were the same with the delayed estimation task, as well as the chosen orientation values. During the probe, a probed orientation appeared on the screen, and participants were required to make a judgment about whether the probed orientation changed compared with the orientation presented in the same location during the sample period, by pressing either the “F” or “J” key. The response keys were counterbalanced across participants, and the maximum response window was 2 s. The probabilities of orientation change and no change were equal. In the change trials, half of the probed orientation was different from all the orientations during the sample display, and it was 40° away from the target item (clockwise or anticlockwise) to make sure the change was detectable (i.e., “change trials”); the other half of the probed orientation was the same as the orientation located next to the probed location (i.e., “lure trials”). The change detection task consisted of 240 trials for each memory load (i.e., Set Size 6 and Set Size 8). Before and after stimulation, participants completed four blocks of the task, and each block consisted of 60 trials with two memory loads randomly mixed. The tDCS setup was consistent with Experiment 2 (Fig. 6*B*).

#### Code accessibility

For all three of experiments, data and code that support the findings are available on the Open Science Framework at https://osf.io/q84a2/. The code is available as Extended Data [noted that modeling was referred to previous work, please see Bays et al., (2009) and Schneegans and Bays (2016)].

#### Data analysis

*Estimation of behavioral performance and tDCS effects*. First, we calculated overall RT and accuracy under each memory load based on all trials. RT was defined as the duration between the onset of the probe and button-press response, and accuracy referred to the proportion of correctly response trials out of all trials. Then, we also obtain RT and accuracy in no change trials, lure trials, and change trials, respectively. In lure trials, higher accuracy indicated better feature binding ability and lower nontarget responses (e.g., lower *p*NT in Experiments 1 and 2). To examine the tDCS effects on each behavioral parameter, we performed a series of 2 (stimulated region, P4 vs sham) × 2 (test time, pre vs post) × 2 (memory load, Set Size 6 vs Set Size 8) ANOVAs. If their interactions were significant, we further analyzed tDCS effects on each memory load. Besides, memory strategy scores were also collected in each set size. We replicated the results that the higher remember-subset strategy scores in Set Size 8 than that in Set Size 6 [SS6, M (SD), 3.250 (1.110); SS8, M (SD), 2.536 (1.071); difference, *t*_(27)_ = 3.487; *p* = 0.0017; BF = 21.319], as well as the correlation between them (*r* = 0.5604; *p* = 0.0017; BF = 8.558). If significant tDCS effect was found, similar correlation analysis would be conducted. Based on the findings of Experiments 1 and 2, the effects of tDCS over PPC on overall RT and accuracy in lure trials were of most interest.

## Results

### Experiment 1

#### PPC stimulation prolonged recall RT

**Figure 2.**
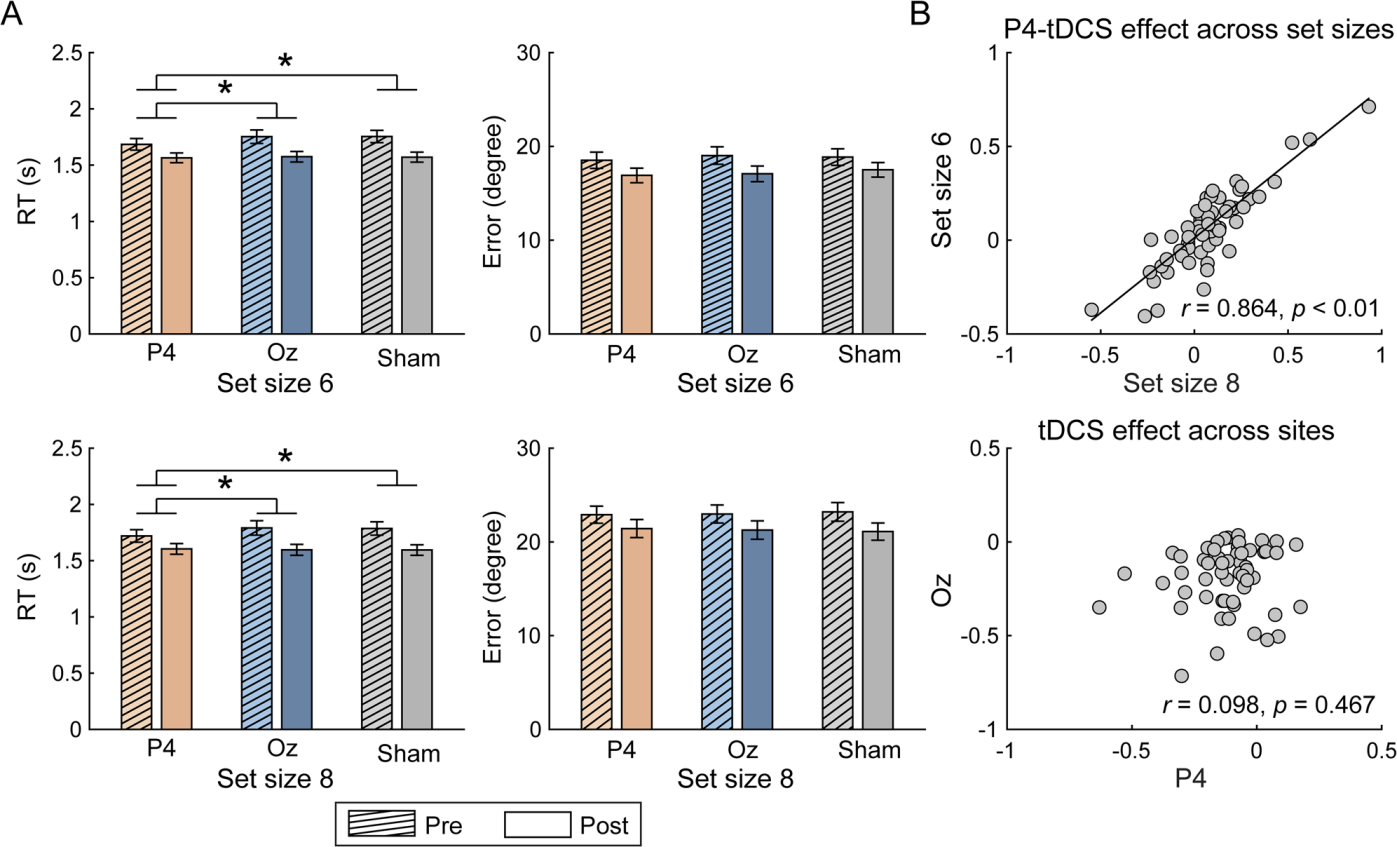
***A***, Changes in RT and recall error across stimulation conditions and memory loads. The error bars represent standard error of the mean (SEM). Statistical test markers indicate significant stimulated region × test time interaction. *p < 0.05. ***B***, Correlation of tDCS effect over PPC across set sizes (top) and tDCS effect over regions (bottom).

For tDCS effects over PPC in RT, the interaction effect of stimulation condition, test time, and memory load was not significant (*F*_(1,56)_ = 0.595; *p* = 0.444; *η_p_*^2^ = 0.011; BF_10_ = 0.205), whereas the interaction between stimulation condition and test time was significant (*F*_(1,56)_ = 6.228; *p* = 0.016; *η_p_*^2^ = 0.100; BF_10_ = 3.366). For both memory loads, we observed significant interactions between stimulation condition and test time (*F*s > 5.179; *p*s < 0.027; *η*_p_^2^s > 0.085; BF_10_s > 2.309). Simple effect tests revealed that, although both PPC stimulation and sham stimulation lead to significantly reduced RT (*t*s > 5.406; *p*s < 0.001; Cohen's *d*s > 0.716; BFs_10_ > 1,000), the decrease was smaller in tDCS over PPC compared with that in sham (i.e., a relatively longer RT after tDCS over PPC; Fig. 2*A*, left). Meanwhile, at the individual level, the tDCS effects on prolonged RTs between two memory loads were highly correlated (*r*_(55)_ = 0.864; *p* < 0.001; BF_10_ > 1,000; Fig. 2*B*, top). Thus, we averaged RT changes in two memory loads to index the tDCS effects on RT in the following analysis. For tDCS effect over OCC in RT, on the contrary, our results revealed no three-way nor two-way interactions between test time and stimulation conditions (*F*s < 2.622; *p*s > 0.111; *η*_p_^2^s < 0.045; BF_10_s < 0.458).

More importantly, we also found significant interactions between two stimulation conditions (i.e., PPC and OCC) and test time for both memory loads (*F*s < 5.969; *p*s < 0.038; *η*_p_^2^ s < 0.096; BF_10_s < 4.908). Similarly, further analyses found that the RT reduction was significantly smaller after PPC stimulation (Set Size 6, *t*_(56)_ = 6.071; *p* < 0.001; Cohen's *d* = 0.804; BF_10_ > 1,000; Set Size 8, *t*_(56)_ = 5.406; *p* < 0.001; Cohen's *d* = 0.716; BF_10_ > 1,000) than that after OCC stimulation (Set Size 6, *t*_(56)_ = 8.179; *p* < 0.001; Cohen's *d* = 1.083; BF_10_ > 1,000; Set Size 8, *t*_(56)_ = 7.810; *p* < 0.001; Cohen's *d* = 1.034; BF_10_ > 1,000). However, our results revealed that effect sizes of tDCS over PPC and OCC were not correlated across subjects (*r*_(55)_ = 0.098; *p* = 0.467; BF_10_ = 0.214; Fig. 2*B*, bottom). Together, these results indicated that PPC stimulation selectively prolonged RT.

Meanwhile, we did not find any significant interaction between test time and stimulation condition for tDCS effects on recall errors (*F*s < 2.202; *p*s > 0.143; *η*_p_^2^ s < 0.038; BF_10_s < 0.218; Fig. 2*A*, right), suggesting no overall VWM performance changes after PPC or OCC stimulation.

#### PPC stimulation increased nontarget responses

**Figure 3.**
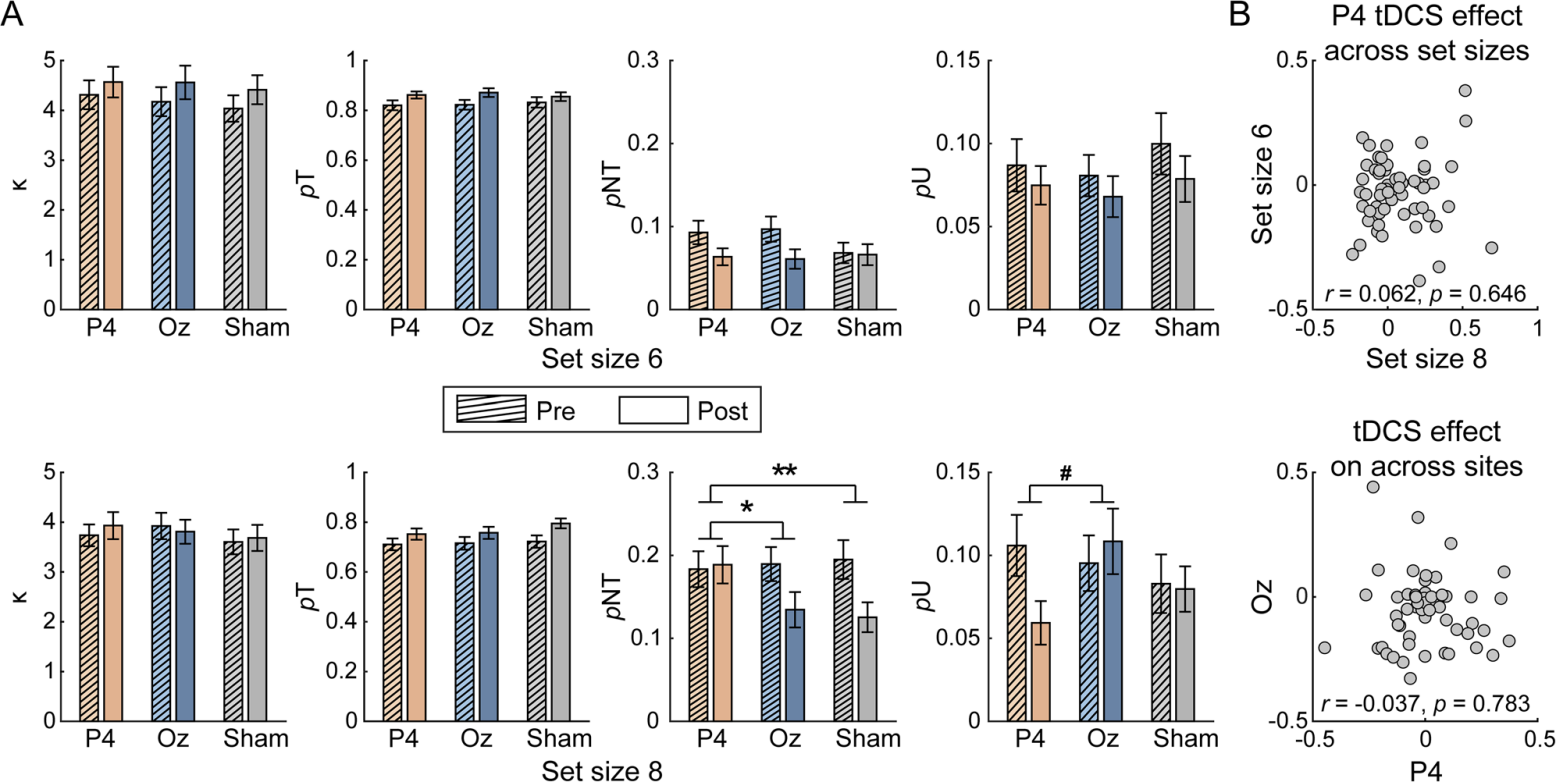
***A***, Changes in response precisions (κ), probability of target responses (*p*T), probability of nontarget responses (*p*NT), and probability of guessing (*p*U) across different stimulation conditions and memory loads. The error bars represent SEM. Statistical test markers indicate significant stimulated region × test time interaction. ^#^0.05 < *p* < 0.1; *p < 0.05; **p < 0.01. ***B***, Correlation of tDCS effect over PPC across set sizes (top) and tDCS effect over regions (bottom).

For tDCS effects over PPC on *p*NT, we found significant interaction among stimulation condition, stimulation time, and memory load (*F*_(1,56)_ = 10.226; *p* = 0.002; *η_p_*^2^ = 0.154; BF_10_ = 4.925). Follow-up two–way ANOVA showed that the interaction between the stimulated region and test time was only significant in Set Size 8 condition (*F*_(1,56)_ = 7.593; *p* = 0.008; *η_p_*^2^ = 0.119; BF_10_ = 3.159; in Set Size 6, *F*_(1,56)_ = 2.157; *p* = 0.147; *η_p_*^2^ = 0.037; BF_10_ = 0.473; Fig. 3*A*). For Set Size 8, nontarget responses were comparable before and after PPC stimulation (*t*_(56)_ = 0.254; *p* = 0.801; Cohen's *d* = 0.034; BF_10_ = 0.149) but became significantly lower after sham stimulation (*t*_(56)_ = 3.426; *p* = 0.001; Cohen's *d* = 0.454; BF_10_ = 24.009). Unlike the tDCS effects on RTs, the effect sizes of tDCS over PPC across memory loads were not correlated (*r*_(55)_ = 0.062; *p* = 0.646; BF_10_ = 0.183; Fig. 3*B*, top).

To further examine the specificity of the tDCS effects of PPC on *p*NT, we compared *p*NT changes before and after stimulation over PPC and OCC in Set Size 8. The interaction between the stimulated region and test time was significant (*F*_(1,56)_ = 4.455; *p* = 0.039; BF_10_ = 2.319). Specifically, comparable *p*NTs were observed after PPC stimulation (*t*_(56)_ = 0.254; *p* = 0.801; Cohen's *d* = 0.034; BF_10_ = 0.149), whereas decreased *p*NTs were found after OCC stimulation (*t*_(56)_ = 2.944; *p* = 0.005; Cohen's *d* = 0.390; BF_10_ = 6.563). Similarly, there was no correlation between the tDCS effects over PPC and OCC on *p*NT (*r*_(55)_ = −0.037; *p* = 0.783; BF_10_ = 0.172; Fig. 3*B*, bottom). Besides, additional correlation analysis revealed that tDCS effects on RT and *p*NT were also independent (*r*_(55)_ = 0.073; *p* = 0.589; BF_10_ = 0.191). Together, these results suggested that, compared with sham stimulation, PPC stimulation specifically increased nontarget responses in the high memory load.

Besides, for tDCS effects over PPC on precisions, target probability, and guessing probability, we only found a marginal three-way interaction effect among the stimulated region, test time, and memory load in *p*U (*F*_(1,56)_ = 3.244; *p* = 0.077; *η*_p_^2^ = 0.055; BF_10_ = 1.542; others, *F*s < 2.732; *p*s > 0.104; *η*_p_^2^ s < 0.047; BF_10_s < 0.159). Further analysis indicated that, in Set Size 8, PPC stimulation decreased the random guesses (*t*_(56)_ = 2.535; *p* = 0.014; Cohen's *d* = 0.336; BF_10_ = 2.670) while no such difference in sham stimulation (*t*_(56)_ = 3.426; *p* = 0.001; Cohen's *d* = 0.454; BF_10_ = 24.009). In contrast, neither three-way interactions nor two-way interactions were observed for OCC stimulation (*F*s < 2.862; *p*s > 0.434; BF_10_s < 0.504), showing no tDCS effect over OCC on all fitting parameters. Together, our results suggested that tDCS over PPC specifically increased the nontarget response while decreasing the random guessing, without changing general VWM performance.

#### tDCS effects over PPC were not modulated by capacity or strategy

**Figure 4.**
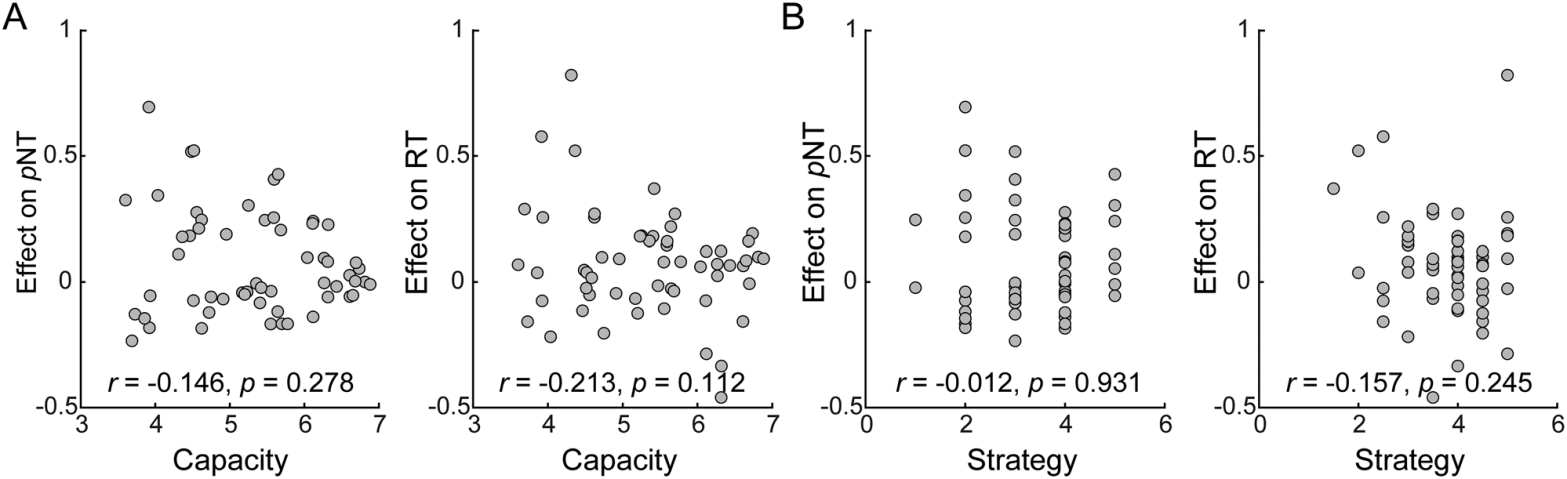
***A***, Correlation between memory capacity and tDCS effects. ***B***, Correlation between memory strategy and tDCS effects. Note that effects on *p*NT were calculated under Set Size 8; effect on RT was averaged across two memory loads.

Correlation results showed that individual capacity or the recall strategy index were not correlated with the PPC tDCS effects of prolonged RT or increased *p*NT (*r*s < −0.213; *p*s > 0.112; BF_10_s < 0.573; Fig. 4).

#### Summary of Experiment 1

Experiment 1 revealed that PPC stimulation specifically prolonged RT and increased *p*NT in the delayed estimation task, supporting the view that the parietal lobe played a critical role in content–context binding during VWM (Gosseries et al., 2018; Cai et al., 2020). In contrast, although studies reported OCC played an important role in information representation during VWM (Bettencourt and Xu, 2016), stimulation over the occipital lobe did not change VWM performance. The results suggested the higher brain areas may play a more causal role in VWM.

By introducing additional metacognition estimates, Pratte (2019) has proposed two types of nontarget responses: the misbinding trials where individuals reported high recall confidence (i.e., “remember trials”), and the informed guessing trials where individuals reported low confidence (i.e., “forgotten trials”). Researchers claimed that these two types of nontarget responses reflected different cognitive and metacognitive processes (Huang, 2020; Mallett et al., 2022). Thus, in Experiment 2, we further examined whether PPC stimulation equally affected these two types of nontarget responses.

### Experiment 2

#### PPC stimulation prolonged recall RT and increased nontarget responses

Consistent with Experiment 1, we observed a significant interaction between stimulation condition and test time on RT (*F*_(1,28)_ = 5.894; *p* = 0.028; *η*_p_^2^ = 0.174; BF_10_ = 5.178). Post hoc tests revealed that the RT decrease after PPC stimulation (*t*_(28)_ = 4.563; *p*s < 0.001; Cohen's *d* = 0.847; BF_10_ = 282.944) was significantly smaller than that after sham stimulation (*t*_(28)_ = 7.356; Cohen's *d* = 1.366; *p*s < 0.001; BF_10_ > 1,000). For *p*NT, the interaction between stimulation condition and test time was also significant (*F*_(1,28)_ = 4.498; *p* = 0.045, *η*_p_^2^ = 0.138; BF_10_ = 2.691). Post hoc analyses revealed that there was no difference after PPC stimulation (*t*_(28)_ = 0.836; *p* = 0.410; Cohen's *d* = 0.155; BF_10_ = 0.272) but a significant decrease in *p*NT after sham stimulation (*t*_(28)_ = 3.816; *p* < 0.001; Cohen's *d* = 0.709; BF_10_ = 46.613). Meanwhile, tDCS effects over PPC on RT and *p*NT were independent across participants (*r*_(27)_ = −0.256; *p* = 0.180; BF_10_ = 0.544), and neither tDCS effect was correlated with individual differences in capacity or strategy [*r*s < −0.297; *p*s > 0.118; BF_10_s < 0.739; capacity, M (SD), 5.497 (1.477); strategy score, M (SD), 3.455 (1.121)]. Besides, similar with the findings in Experiment 1, we observed a similar numerical trend of two-way interaction between stimulated region and test time for *p*U (*F*_(1,28)_ = 2.780; *p* = 0.107; *η*_p_^2^ = 0.090; BF_10_ = 1.497), while there was no significant effects on recall error, confidence ratings, and other parameters (*F*s < 0.978; *p*s > 0.331; *η*_p_^2^s < 0.034; BF_10_s < 0.403).

#### tDCS effects over PPC on RT were greater in forgotten trials than in remembered trials

**Figure 5.**
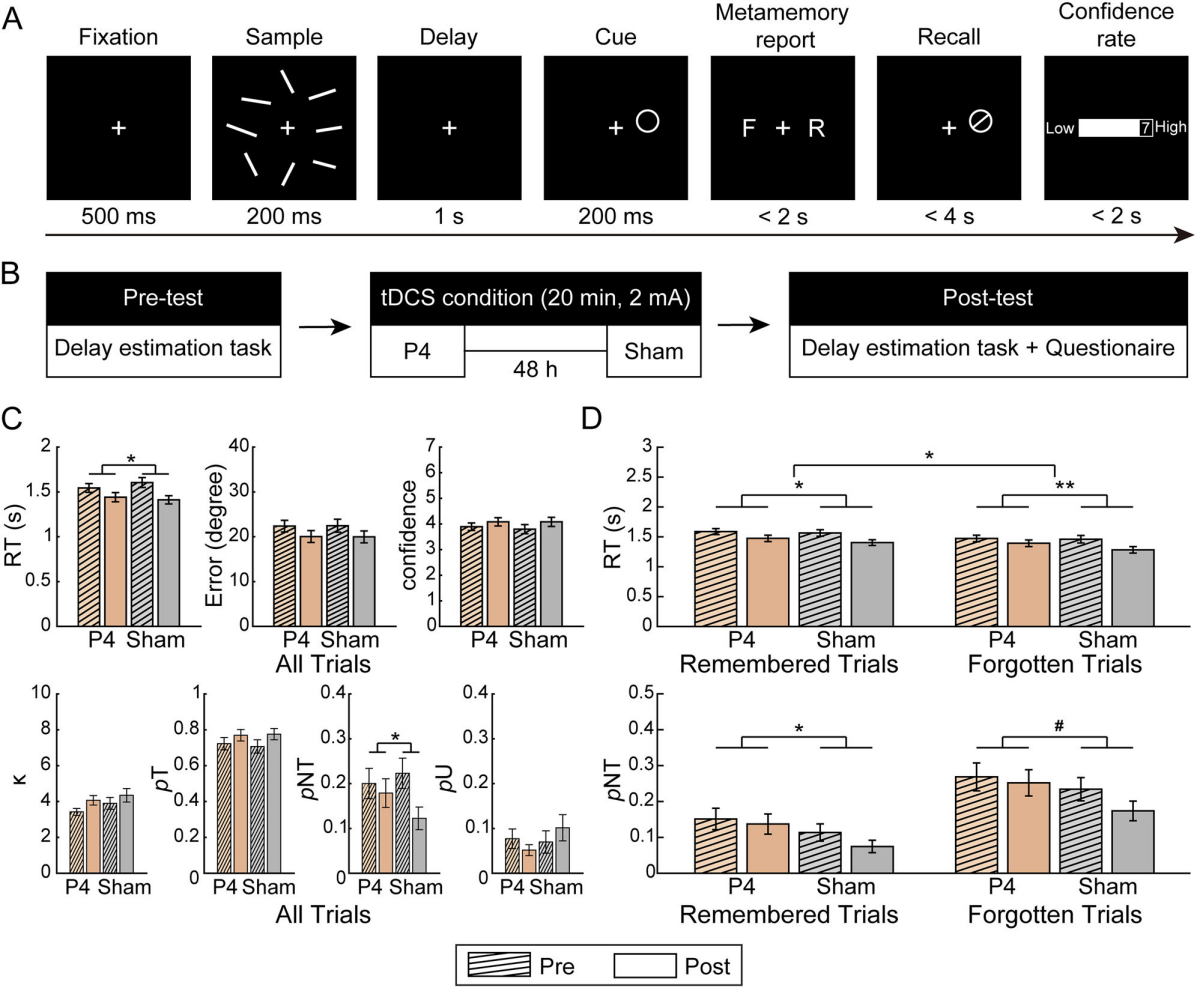
***A***, Schematic diagrams of the delay estimation task. ***B***, tDCS procedures. ***C***, Changes in RT, error, confidence rating, and fitting parameters across stimulation conditions. The error bars represent SEM. Statistical test markers indicate significant interactions. ^#^0.05 < *p* < 0.1; *p < 0.05; **p < 0.01. ***D***, Changes in RT and *p*NT for remembered and forgotten trials across stimulation conditions.

We further examined the tDCS effects over PPC between forgotten and remembered trials. For RT, the interaction among trial type, stimulation condition, and test time were significant (*F*_(1,28)_ = 6.385; *p* = 0.017; *η_p_*^2^ = 0.186; BF_10_ = 2.372; Fig. 5*D*, top). Further two-way repeated–measure ANOVAs for each trial type indicated that the effect size in forgotten trials (*F*_(1,28)_ = 11.506; *p* = 0.002; *η*_p_^2^ = 0.291; BF_10_ = 65.871) was larger than in remembered trials (*F*_(1,28)_ = 4.293; *p* = 0.048; *η*_p_^2^ = 0.133; BF_10_ = 2.244). For *p*NT, however, no significant interaction effect among trial type, stimulation condition, and test time were observed (*F*s < 2.707; *p*s > 0.111; *η*_p_^2^s < 0.088; BF_10_s < 0.225; Fig. 5*D*, bottom), indicating comparable tDCS effects in two types of nontarget trials.

#### Summary of Experiment 2

In Experiment 2, we replicated the main findings of Experiment 1 that PPC stimulation increased RT and *p*NT during VWM. More importantly, compared with remembered trials, tDCS effects on RT were greater in forgotten trials while were comparable on *p*NT. In sum, these results suggested that PPC was causally involved in two types of nontarget responses, while it may be through different mechanisms.

A recent computational modeling study indicated that VWM recall and recognition involved different cognitive processes (Kahana, 2020). Since delayed estimation and change detection are two typical VWM tasks to estimate memory recall and recognition respectively, we further examined whether the tDCS over PPC mainly affected VWM retrieval processes and caused behavioral changes, by using a change detection task in Experiment 3.

### Experiment 3

#### No tDCS effect over PPC on RT or misbinding processes in recognition

**Figure 6.**
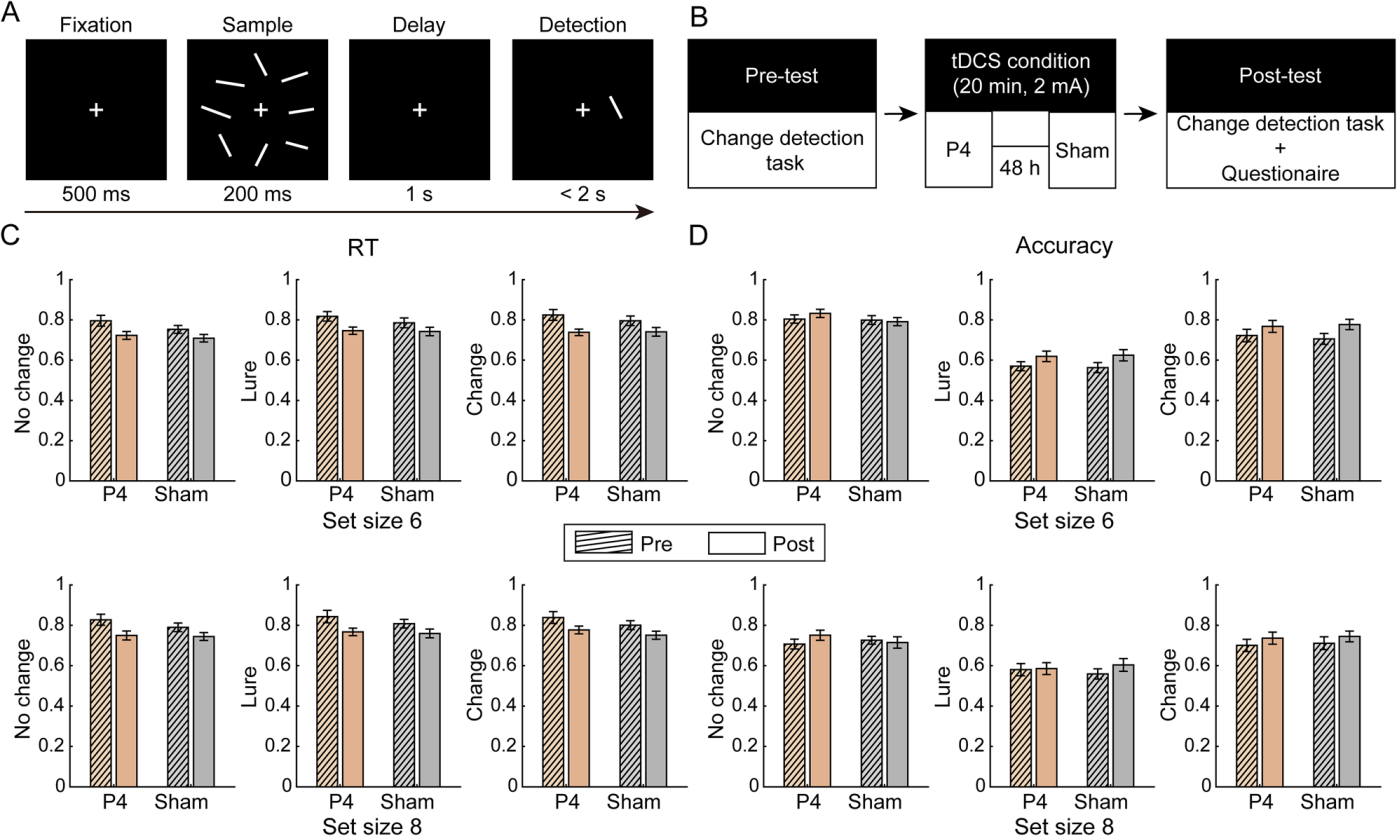
***A***, Schematic diagrams of the change detection task. ***B***, tDCS procedures. Changes in RT (***C***) and accuracy (***D***) across trial types and stimulation conditions. The error bars represent SEM.

No interaction effect between stimulation and test time was significant for RT (*F*s < 1.251; *p*s > 0.273; *η*_p_s^2^ < 0.044; BF_10_s < 0.390) or for accuracy in lure trials (*F*s < 0.832; *p*s > 0.370; BF_10_s < 0.391), indicating that PPC stimulation did not affect RT or binding processes in VWM recognition (Fig. 6*C*,*D*). Similarly, there was no significant interaction effect on other behavioral parameters (*F*s < 1.235; *p*s > 0.246; *η*_p_s^2^ < 0.042; BF_10_s < 0.586; except for a trend effect on accuracy in high memory load in no change trials, *F*_(1,27)_ = 3.441; *p* = 0.075; BF_10_ = 0.945).

## Discussion

The current study established the causal relationship between posterior parietal activity and feature binding during VWM retrieval through three tDCS experiments. First, we found that anodal tDCS over PPC selectively increased recall RT as well as nontarget responses in the delayed estimation task. Meanwhile, combined with metacognitive evaluations, we clarified these tDCS effects could be observed in both types of nontarget responses (i.e., misbinding and informed guessing). Besides, we further identified that the effects of tDCS over PPC were specific during memory retrieval by demonstrating that such effects were not observed during memory recognition. Together, our findings deepen our understanding of the involvement of PPC in the feature binding during VWM retrieval.

First of all, in two independent samples, we replicated that anodal tDCS over PPC increased nontarget responses in the delayed estimation task. These results supported recent fMRI studies that emphasized the close relationship between posterior parietal activity and the feature binding processes during VWM (Gosseries et al., 2018; Cai et al., 2020). Meanwhile, we noticed the increased nontarget responses along with a trend of decreased random guesses. That is, tDCS over PPC biased the recall process. Two possibilities could explain these findings. One possibility is that enhanced posterior parietal activity may facilitate the reinstatement of content information from multiple items during retrieval, leading to an increased probability of nontarget responses. Supporting this view, Baddeley (2000) proposed the posterior parietal lobe as a core area for the episodic buffer during WM, and Xie et al. (2017) further interpreted PPC as a hub of multisensory information integration, which claimed PPC's critical role in representing and combining different features. Moreover, our results emphasized that PPC was causally involved during memory retrieval instead of recognition. Comparing the delayed estimation and change detection tasks, previous studies have shown similar neural activity during WM maintenance. For instance, EEG studies have found similar contralateral delay activity (Vogel and Machizawa, 2004; Adam et al., 2018; Cai et al., 2022), and fMRI studies showed similar frontoparietal activity during these two tasks (Cai et al., 2018; Kim, 2019). In contrast, during the response, a recent study suggested that the delayed estimation task is mainly based on detailed retrieval while the change detection task is based on familiarity judgment (Kahana, 2020). Although few studies directly compared the PPC activity between recall and recognition during WM, considerable evidence has supported the critical role of PPC in episodic memory retrieval but not in recognition. For example, studies have revealed that both activation strength (Wagner et al., 2005; Cabeza et al., 2008; Sestieri et al., 2017) and the neural representations in PPC (Xiao et al., 2017) increased during memory retrieval but not during recognition (Dobbins et al., 2003). Currently, our findings confirmed the causal relationship between the PPC and retrieval during VWM, which was similar to those in episodic memory.

Another possibility proposed that enhanced posterior parietal activity may increase cognitive resources and lead to the adoption of a more proactive retrieval strategy, increasing nontarget responses. Our results revealed that tDCS over PPC comparably increased both types of nontarget responses (i.e., misbinding and informed guessing). Researchers have proposed that misbinding responses were generated when participants misorganized information across different items, while informed guessing reflected participants actively making choices from all the memorized information (Pratte, 2019). Consistently, recent studies revealed that misbinding mainly resulted from less efficient information processing during early encoding or storage (Emrich and Ferber, 2012; Zokaei et al., 2014; Pertzov et al., 2017) whereas informed guessing more likely reflected the different neural activity during late storage or memory retrieval (Pratte, 2019; Huang, 2020). Consequently, both nontarget responses required more cognitive effort compared with random guesses. From the source-consumption perspective, the latest study revealed that parietal tDCS could reduce the cumulative fatigue effect during tasks (Hemmerich et al., 2023). Therefore, we could not exclude the possibility that anodal PPC stimulation enabled individuals to generate both more proactive nontarget responses during VWM retrieval.

Besides, we also found that PPC stimulation prolonged recall RT, which was in line with both explanations about increased nontarget responses above. However, some evidence in the current study suggested that the tDCS over PPC affected recall RT and feature binding through different pathways. For example, we found that tDCS over PPC only increased nontarget responses under high memory load, whereas it changed RTs across memory loads, and the effects were highly correlated across loads at the individual level. Meanwhile, we observed greater tDCS effects on RTs in informed guessing trials than in misbinding trials, while comparable effects on nontarget responses between these two types of trials. Furthermore, tDCS effects on RTs and nontarget responses were always independent at the individual level. Supporting these findings, the latest meta-analysis demonstrated that different PPC stimulation patterns enhanced WM accuracy and RT separately (i.e., different frequencies; Wischnewski et al., 2024). However, future studies are needed to further clarify how PPC is differently involved in the recall speed and accuracy of memory retrieval.

To be noted, our findings were not consistent with some previous relevant studies. Regarding the tDCS effects on increasing the nontarget response during the delayed estimation task, for example, using a similar paradigm, another study found anodal PPC stimulation did not change the nontarget response rate but increased the recall precision. On the opposite, the PPC cathodal stimulation decreased the nontarget response rate and increased the target response rate and recall precision (Heinen et al., 2016). In this study, the reversed tDCS effect of cathodal stimulation on the nontarget response rate could be well explained by the polarity effects of tDCS reported in accumulating studies (Nitsche and Paulus, 2000; Stagg and Nitsche, 2011) and supported the causality between PPC activities and feature bindings observed in the current study. Meanwhile, we would attribute the inconsistent anodal stimulation effects across studies to differences in timings of stimulations as well as task difficulty. The tDCS in our study was conducted when participants took a rest between tasks while the stimulation in Heinen's study was conducted exactly during the tasks. According to the recent state-dependent theory, the neural modulation effects were largely dependent on the ongoing neural states (Bradley et al., 2022). Besides, the tDCS effects in our study were observed in a supra-capacity condition (Set Size 8), while tDCS effects in Heinen's study were in a much lower, around-capacity condition (Set Size 4). According to previous studies, task difficulty could be a critical modulation factor influencing the tDCS effects (Pope and Miall, 2012; Vergallito et al., 2022). Given that participants were more likely to employ the remember-subset strategy in the high-load condition, it is plausible that tDCS may have specifically impacted cognitive processes underlying this memory strategy (e.g. suppression “distractors” beyond memory capacity). In addition, we did not find that tDCS over PPC improved the general VWM performance in either delayed estimation or change detection tasks, which was consistent with some recent studies (Heinen et al., 2016; Robison et al., 2017; Jiang et al., 2024) but challenged some other earlier studies (Li et al., 2017; Wang et al., 2019). Besides, we did not replicate the correlation between tDCS effects and individual capacity (Tseng et al., 2012; Hsu et al., 2014) or the adoption of remember-subset strategy (Wang et al., 2020). Usually, as we mentioned above, researchers suggested that these differences could be generally explained by a series of detailed methodological settings. However, regarding our null tDCS effects in the change detection task, in particular, we also suggested to understand these differences from the different probed displays and stimulus type. For example, Tseng et al. (2012) used a change detection task with the whole-item comparison design while we used the single-item comparison. Previous studies have revealed that the change detection judgments on the whole-item display can depend on additional integrated information (such as the general configuration and relationships between items) while single-item judgment cannot (Morey, 2011; Rouder et al., 2011). Thus, the tDCS over PPC may only facilitate processes for more integrated information instead of single-item retrieval. Meanwhile, we cannot exclude the differences was caused by varying stimuli across studies given there are not a few studies have revealed dissociated cognitive and neural basis underlying VWMs for different stimulus types (e.g., color vs orientation; Jackson et al., 2011; Huang, 2015).

Different from the PPC stimulation effects, it is noteworthy that we did not observe any tDCS effects over the occipital cortex on VWM. A set of recent studies found there was a location-specific neural representation in the occipital cortex which requires accurate item–context binding (Fulvio et al., 2023; Teng and Postle, 2024), and some other studies also reported these neural representations predicted nontarget responses at the individual level (van Lamsweerde and Johnson, 2015; Cai et al., 2020). We suggested two possible explanations for this inconsistency. First, unlike the sustained activation of PPC during maintenance, the neural representations of the item or its context information did not depend on sustained activation in the occipital cortex (Harrison and Tong, 2009; Riggall and Postle, 2012). Since tDCS is expected to increase the neural activity of specific areas instead of promoting the neural representation directly, tDCS over the occipital cortex could not significantly affect behavior. Second, previous studies have suggested that occipital neural activity is regulated by feedback signals from the frontal and posterior parietal regions (Halgren et al., 2002). Therefore, even if the occipital neural representation is affected by tDCS, the occipital cortex could maintain the memory information efficiently by receiving feedback signals from other brain areas. Together, these results indicated that the occipital activity has no causal effect on VWM performance or feature binding process.

Finally, some limitations need to be paid attention in the current study. First, our results were not consistent with some relevant studies mentioned above, and we cannot identify whether these differences are attributed to some specific factors (i.e., task paradigm, difficulty, stimulus type, individual differences, timing of stimulation, etc.) or a more complex interaction effect between them. Although our findings may not end the existing controversies, our study strongly reminded that future studies should systematically explore the potential factors influencing tDCS effects, and combining the high-definition stimulations and neuroimaging methods could provide insightful views. Second, we found that single-session tDCS only changed the response bias for nontargets but failed to change overall working memory performance, which should be further explored in future studies. For example, recent studies have demonstrated that local brain oscillations and interarea synchronizations contributed to feature binding during VWM (Barbosa et al., 2019; Zhang et al., 2019); thus future studies using transcranial alternative current stimulation (tACS) to change these band-specific neural activities may better improve the binding efficiency and VWM performance. Meanwhile, recent studies have also demonstrated that high-frequency randomized noise stimulation reveals a stronger effect on changing the cortical activities, which could also be a potential way to improve the general VWM performance (Terney et al., 2008; Murphy et al., 2020).

In conclusion, the present study demonstrates that enhanced posterior parietal activity prolongs RT in VWM retrieval and increases the probability of binding errors, and these effects are observed in two types of binding errors (i.e., misbinding and informed guessing). Our findings provide direct evidence of the causal relationship between the PPC and feature binding, deepening our understanding of the neural basis of feature binding in VWM.

### Data and code availability statement

Data and code that support the findings of this study are available on the Open Science Framework at https://osf.io/q84a2/.

10.1523/ENEURO.0265-24.2024.d1 Extended Data Noted that modeling was referred to previous work, please see Bays et al. (2009) and Schneegans and Bays (2016). Download Extended Data, ZIP file.

## Synthesis

Reviewing Editor: Frederike Beyer, Queen Mary University of London Decisions are customarily a result of the Reviewing Editor and the peer reviewers coming together and discussing their recommendations until a consensus is reached. When revisions are invited, a fact-based synthesis statement explaining their decision and outlining what is needed to prepare a revision will be listed below. The following reviewer(s) agreed to reveal their identity: Valentina Pergher, Klaartje Heinen.

Reviewer 1 comments

The authors investigated the causal relationship between posterior parietal cortex (PPC) and feature binding during visual working memory (VWM), performing three transcranial direct current stimulation (tDCS) experiments.

The manuscript is well written, the methods are well reported and can be generalized to other experiments, and the results very clear. However, there are a few items that need to be addressed.

Abstract

- I suggest adding a few more sentences in the conclusions.

Introducction

- There are some missing references through the manuscript while gicing the deinition of WM or other topics

- I suggest adding a reacher explanation about tDCS, what it is, its mechanisms, and so on.

Methods

- I suggest adding an explanation about the choice of the current (2mA) use for the stimulation

- Same for the choice of the cognitive task used for the experiments.

Discussion

- I suggest adding more interpretation about the authors' findings and connections between the current fidnings with previous literature studies

- I agee that one limitation of the study is that the authors could have tested groups using different stimulation techniches, such as tACS or tRNS.

Reviewer 2 comments

The current paper presents a study, in which the authors probed the causal role of the right PPC and Occipital Cortex in feature binding during VWM. This was investigated using a delayed recall paradigm, that allowed the dissociation of distinct aspects of visual working memory, such as precision (variability in memory for target feature), misbinding (reporting of a non-target feature) and random guessing. In addition to this, RT's were measured.

In 3 experiments the authors investigated the following questions:

-Is the right PPC causally and specifically implied in feature binding (exp I).

-If yes, Is a potential influence due to misbinding (during remembered trials) or informed guessing (during forgotten trials)?

-Does this effect occur during WM recall or recognition?

The results show that anodal tDCS to right PPC increases response times during delayed recall and leads to an increase in non-target responses (Exp.I). Interestingly, the latter effect was set-size dependent and only present in the high-load (8 items) condition.

The second experiment used metacognitive measures to dissociate between two types of non-target responses. The results suggest that the tDCS induced increase in RT mainly reflected informed guessing (during forgotten trials), while an increase in non-target responses was due to both misbinding (remembered trials) and informed guessing (forgotten trials).

In the third experiment, no effects of right PPC tDCS were observed in a paradigm that specifically probed recognition memory, suggesting that the pPC is specifically involved in recall and not recognition (familiarity).

This is an interesting set of experiments, which are neatly set-up with quite clear results. However, I have several questions about the choice of experimental parameters and interpretations of results/discussion that require some clarification.

-Exp.1: Why did the authors choose around/supra-capacity set-sizes of 6- and 8 items? Is this based on the study by Wang et al (2020)? (p.7 l.111). If I understand correctly they reported a higher chance of null-results when participants are instructed to remember a supra-capacity set-size (as opposed to a sub-set of presented items). It is not clear to me whether the authors found a difference in applied strategy between the two set-sizes in the current study. (p.10 l.182-183). Can the authors clarify these questions?

- While the authors report a null result in terms of general accuracy and precision, their paradigm allows dissection of distinct aspects of VWM, which revealed a decrease in performance in terms of non-target (nT) responses. The fact that this is not reflected in an overall worsening of performance is likely due to a trend in opposite direction for random guesses (pU), which is worthwhile to mention. It would be interesting to compare these results in more detail to those reported by Heinen et al. (2016), who used a similar delayed recall paradigm. In contrast to what is stated in the discussion, this study did not report a mere null-result. While indeed no effect of anodal tDCS to rPPC was detected in their study, a specific effect on pT (increase) and nT errors (decrease) was observed for cathodal tDCS, applied during task-performance. Hence, both studies report a specific and causal role for rPPC in feature binding (nT errors). It would be informative to emphasise this in the manuscript. Several aspects are also important to consider cq. further discuss:

-the importance of 1.timing of- and 2.polarity of stimulation. Why would anodal stimulation pre-task deteriorate feature binding, while cathodal stimulation during the task improves it?

-In addition to this: Eventhough the positive effect of pU (decrease in random errors) is a non-significant trend here, it may be remarked upon that this trend is in accordance to previous findings. Could the authors comment why anodal tDCS may have opposite effects on nT versus random errors?

-Exp. 2: why could non-target responses in 'forgotten' trials not be attributed to misbinding? (p.14 l.269) Can this be clarified a bit more in text?

-Exp. 3: It is not clear to me how large the orientation deviation was in the recognition trial?

In general: these null-results are a bit surprising, as previous studies used change-detection tasks (Tseng et al., 2012)? Can the authors comment on this?

## Author Response

Responses to Reviewers' Comments Both reviewers are happy with the revisions and the improvements made to the manuscript. They do highlight a few minor corrections to be made before the manuscript can go into publication Thank you for these positive feedbacks. Q1. p.4 l.65 Should this not be 'In the mis-binding condition, individuals are UNCONSCIOUS of recalling a non-target item.' ? Thanks for pointing out this typo and we apologize for this mistake. In the revised manuscript, we have corrected this sentence, which reads "In the mis-binding condition, individuals are unconscious of recalling a non-target item" (page 4, line 65). Q2. I think the finding that the recall-strategy differed between memory-load conditions and the 'remember subset'-strategy was more likely in the higher memory-load condition, deserves some more emphasis and elaboration. Especially as the tDCS effect were only observed in this condition (p.9 l.187). Could it be that it is a specific process underlying this strategy that is affected by tDCS, such as (feature binding during) distracter suppression? This could fi. be added to the discussion p.24 l.523-24 Thanks for raising this important question. We agree that the strategy difference between memory loads could indeed offer a potential explanation for the differential tDCS effects, particularly given the higher likelihood of using the remember-subset strategy in the set size 8 condition, where tDCS effects were observed. In our original discussion, we did not emphasize this perspective, largely due to the lack of a significant correlation between the remember-subset strategy score and the amplitude of tDCS effects at the individual level across all three experiments (as noted on page 24, lines 528-529). However, as you pointed out, further elaborating on this issue may provide additional clarity to our findings. In the revised discussion, we have added the following, "Given that participants were more likely to employ the remember-subset strategy in the high-load condition, it is plausible that tDCS may have specifically impacted cognitive processes underlying this memory strategy (e.g., suppression "distractors" beyond memory capacity)" (page 24, lines 526-529). Typo: Q3. p.17 l.357. should this not be 'and other parameters were INSIGNIFICANT'? Thanks for pointing out this typo. We have rephrased this sentence to clarify the results, which reads, "there was no significant tDCS effects on recall error, confidence ratings, and other parameters (Fs < 0.978, ps > 0.331, ηp 2 s < 0.034, BF10s < 0.403)" (page 17, lines 356- 358).

## References

[B1] Adam KCS, Robison MK, Vogel EK (2018) Contralateral delay activity tracks fluctuations in working memory performance. J Cogn Neurosci 30:1229–1240. 10.1162/jocn_a_01233 29308988 PMC6283409

[B2] Ashbridge E, Cowey A, Wade D (1999) Does parietal cortex contribute to feature binding? Neuropsychologia 37:999–1004. 10.1016/S0028-3932(98)00160-2 10468364

[B3] Baddeley A (2000) The episodic buffer: a new component of working memory? Trends Cogn Sci 4:417–423. 10.1016/S1364-6613(00)01538-2 11058819

[B4] Baddeley AD, Hitch G (1974) Working memory. In: *Psychology of learning and motivation* (Bower GH, ed), Vol 8, pp 47–89. New York: Academic Press.

[B5] Barbosa J, Sreenivasan K, Compte A (2019) *Feature-binding in working memory through neuronal synchronization* 2019 conference on cognitive computational neuroscience.

[B6] Bays PM, Catalao RFG, Husain M (2009) The precision of visual working memory is set by allocation of a shared resource. J Vis 9:1–11. 10.1167/9.10.7 19810788 PMC3118422

[B7] Bettencourt KC, Xu Y (2016) Decoding the content of visual short-term memory under distraction in occipital and parietal areas. Nat Neurosci 19:150–157. 10.1038/nn.4174 26595654 PMC4696876

[B8] Bradley C, Nydam AS, Dux PE, Mattingley JB (2022) State-dependent effects of neural stimulation on brain function and cognition. Nat Rev Neurosci 23:459–475. 10.1038/s41583-022-00598-1 35577959

[B9] Braet W, Humphreys GW (2009) The role of reentrant processes in feature binding: evidence from neuropsychology and TMS on late onset illusory conjunctions. Vis Cogn 17:25–47. 10.1080/13506280802193318

[B10] Brainard DH (1997) The psychophysics toolbox. Spat Vis 10:433–436. 10.1163/156856897X00357 9176952

[B11] Cabeza R, Ciaramelli E, Olson IR, Moscovitch M (2008) The parietal cortex and episodic memory: an attentional account. Nat Rev Neurosci 9:613–625. 10.1038/nrn2459 18641668 PMC2692883

[B12] Cai Y, Fulvio JM, Samaha J, Postle BR (2022) Context binding in visual working memory is reflected in bilateral event-related potentials, but not in contralateral delay activity. eNeuro 9:ENEURO.0207-22.2022. 10.1523/ENEURO.0207-22.2022 36265905 PMC9652780

[B13] Cai Y, Fulvio JM, Yu Q, Sheldon AD, Postle BR (2020) The role of location-context binding in nonspatial visual working memory. eNeuro 7:6–ENEURO.0430-20.2020. 10.1523/ENEURO.0430-20.2020 33257529 PMC7773890

[B14] Cai Y, Urgolites Z, Wood J, Chen C, Li S, Chen A, Xue G (2018) Distinct neural substrates for visual short-term memory of actions. Hum Brain Mapp 39:4119–4133. 10.1002/hbm.24236 29947094 PMC6866292

[B15] Dobbins IG, Rice HJ, Wagner AD, Schacter DL (2003) Memory orientation and success: separable neurocognitive components underlying episodic recognition. Neuropsychologia 41:318–333. 10.1016/S0028-3932(02)00164-1 12457757

[B16] Dumont R, Majerus S, Hansenne M (2021) Transcranial direct current stimulation (tDCS) over the intraparietal sulcus does not influence working memory performance. Psychol Belg 61:200–211. 10.5334/pb.534 34277028 PMC8269793

[B17] Emrich SM, Ferber S (2012) Competition increases binding errors in visual working memory. J Vis 12:12. 10.1167/12.4.12 22523399

[B18] Faul F, Erdfelder E, Lang A-G, Buchner A (2007) G*Power 3: a flexible statistical power analysis program for the social, behavioral, and biomedical sciences. Behav Res Methods 39:175–191. 10.3758/BF03193146 17695343

[B19] Fulvio JM, Yu Q, Postle BR (2023) Strategic control of location and ordinal context in visual working memory. Cereb Cortex 33:8821–8834. 10.1093/cercor/bhad164 37164767 PMC10321086

[B20] Gong M, Li S (2014) Learned reward association improves visual working memory. J Exp Psychol 40:841–856. 10.1037/a0035131 24392741

[B21] Gosseries O, Yu Q, LaRocque JJ, Starrett MJ, Rose NS, Cowan N, Postle BR (2018) Parietal-occipitalinteractions underlying control- and representation-related processes in working memory for nonspatial visual features. J Neurosci 38:4357–4366. 10.1523/JNEUROSCI.2747-17.2018 29636395 PMC5932644

[B22] Halgren E, Boujon C, Clarke J, Wang C, Chauvel P (2002) Rapid distributed fronto-parieto-occipital processing stages during working memory in humans. Cereb Cortex 12:710–728. 10.1093/cercor/12.7.710 12050083

[B23] Harrison SA, Tong F (2009) Decoding reveals the contents of visual working memory in early visual areas. Nature 458:632–635. 10.1038/nature07832 19225460 PMC2709809

[B24] Heinen K, Sagliano L, Candini M, Husain M, Cappelletti M, Zokaei N (2016) Cathodal transcranial direct current stimulation over posterior parietal cortex enhances distinct aspects of visual working memory. Neuropsychologia 87:35–42. 10.1016/j.neuropsychologia.2016.04.028 27143222 PMC4915336

[B25] Hemmerich K, Lupiáñez J, Luna FG, Martín-Arévalo E (2023) The mitigation of the executive vigilance decrement via HD-tDCS over the right posterior parietal cortex and its association with neural oscillations. Cereb Cortex 33:6761–6771. 10.1093/cercor/bhac540 36646467

[B26] Hsu TY, Tseng P, Liang WK, Cheng SK, Juan CH (2014) Transcranial direct current stimulation over right posterior parietal cortex changes prestimulus alpha oscillation in visual short-term memory task. Neuroimage 98:306–313. 10.1016/j.neuroimage.2014.04.069 24807400

[B27] Huang L (2015) Color is processed less efficiently than orientation in change detection but more efficiently in visual search. Psychol Sci 26:646–652. 10.1177/0956797615569577 25834029

[B28] Huang L (2020) Distinguishing target biases and strategic guesses in visual working memory. Atten Percept Psychophys 82:1258–1270. 10.3758/s13414-019-01913-2 31758526

[B29] Jackson MC, Morgan HM, Shapiro KL, Mohr H, Linden DE (2011) Strategic resource allocation in the human brain supports cognitive coordination of object and spatial working memory. Hum Brain Mapp 32:1330–1348. 10.1002/hbm.21112 20715083 PMC3326378

[B30] Jiang S, Jones M, von Bastian CC (2023) Mechanisms of cognitive change: training improves the quality but not the quantity of visual working memory representations. J Cogn 6:42. 10.5334/joc.306 37483542 PMC10360971

[B31] Jiang S, Jones M, von Bastian CC (2024) TDCS over PPC or DLPFC does not improve visual working memory capacity. Commun Psychol 2:20. 10.1038/s44271-024-00067-8 39242793 PMC11332112

[B32] Kahana MJ (2020) Computational models of memory search. Annu Rev Psychol 71:107–138. 10.1146/annurev-psych-010418-103358 31567043 PMC8389167

[B33] Kim H (2019) Neural activity during working memory encoding, maintenance, and retrieval: a network-based model and meta-analysis. Hum Brain Mapp 40:4912–4933. 10.1002/hbm.24747 31373730 PMC6865408

[B34] Kirova AM, Bays RB, Lagalwar S (2015) Working memory and executive function decline across normal aging, mild cognitive impairment, and Alzheimer's disease. Biomed Res Int 2015:748212. 10.1155/2015/748212 26550575 PMC4624908

[B35] Lee C, Jung Y-J, Lee SJ, Im C-H (2017) COMETS2: an advanced MATLAB toolbox for the numerical analysis of electric fields generated by transcranial direct current stimulation. J Neurosci Methods 277:56–62. 10.1016/j.jneumeth.2016.12.008 27989592

[B36] Li S, Cai Y, Liu J, Li D, Feng Z, Chen C, Xue G (2017) Dissociated roles of the parietal and frontal cortices in the scope and control of attention during visual working memory. Neuroimage 149:210–219. 10.1016/j.neuroimage.2017.01.061 28131893

[B37] Luck D, Buchy L, Lepage M, Danion J-M (2009) Examining the effects of two factors on working memory maintenance of bound information in schizophrenia. J Int Neuropsychol Soc 15:597–605. 10.1017/S1355617709090833 19573278

[B38] Makovski T, Lavidor M (2014) Stimulating occipital cortex enhances visual working memory consolidation. Behav Brain Res 275:84–87. 10.1016/j.bbr.2014.09.004 25205369

[B39] Mallett R, Lorenc ES, Lewis-Peacock JA (2022) Working memory swap errors have identifiable neural representations. J Cogn Neurosci 34:776–786. 10.1162/jocn_a_01831 35171256 PMC11126154

[B40] Mayer JS, Fukuda K, Vogel EK, Park S (2012) Impaired contingent attentional capture predicts reduced working memory capacity in schizophrenia. PLoS One 7:e48586. 10.1371/journal.pone.0048586 23152783 PMC3495971

[B41] Morey RD (2011) A Bayesian hierarchical model for the measurement of working memory capacity. J Math Psychol 55:8–24. 10.1016/j.jmp.2010.08.008

[B42] Murphy OW, Hoy KE, Wong D, Bailey NW, Fitzgerald PB, Segrave RA (2020) Transcranial random noise stimulation is more effective than transcranial direct current stimulation for enhancing working memory in healthy individuals: behavioural and electrophysiological evidence. Brain Stimul 13:1370–1380. 10.1016/j.brs.2020.07.001 32659482

[B43] Nitsche MA, Paulus W (2000) Excitability changes induced in the human motor cortex by weak transcranial direct current stimulation. J Physiol 527:633–639. 10.1111/j.1469-7793.2000.t01-1-00633.x 10990547 PMC2270099

[B44] Oberauer K, Lin H-Y (2017) An interference model for visual and verbal working memory. Psychol Rev 124:21–59. 10.1037/rev0000044 27869455

[B45] Pertzov Y, Manohar S, Husain M (2017) Rapid forgetting results from competition over time between items in visual working memory. J Exp Psychol 43:528–536. 10.1037/xlm0000328 27668485 PMC5377990

[B46] Pope PA, Miall RC (2012) Task-specific facilitation of cognition by cathodal transcranial direct current stimulation of the cerebellum. Brain Stimul 5:84–94. 10.1016/j.brs.2012.03.006 22494832 PMC3379560

[B47] Pratte MS (2019) Swap errors in spatial working memory are guesses. Psychon Bull Rev 26:958–966. 10.3758/s13423-018-1524-8 30242631 PMC7093911

[B48] Riggall AC, Postle BR (2012) The relationship between working memory storage and elevated activity as measured with functional magnetic resonance imaging. J Neurosci 32:12990–12998. 10.1523/JNEUROSCI.1892-12.2012 22993416 PMC3470886

[B49] Robison MK, McGuirk WP, Unsworth N (2017) No evidence for enhancements to visual working memory with transcranial direct current stimulation to prefrontal or posterior parietal cortices. Behav Neurosci 131:277–288. 10.1037/bne0000202 28714714

[B50] Rouder JN, Morey RD, Morey CC, Cowan N (2011) How to measure working memory capacity in the change detection paradigm. Psychon Bull Rev 18:324–330. 10.3758/s13423-011-0055-3 21331668 PMC3070885

[B51] Salehinejad MA, Ghanavati E, Kuo M-F, Nitsche MA (2023) The role of circadian preferred time of day and sleep pressure in tDCS-induced neuroplasticity and associated cognition. Brain Stimul 16:203–204. 10.1016/j.brs.2023.01.266

[B52] Salehinejad M, Kuo M, Nitsche M (2019) The impact of chronotypes and time of the day on tDCS-induced motor cortex plasticity and cortical excitability. Brain Stimul 12:421. 10.1016/j.brs.2018.12.365

[B53] Schneegans S, Bays PM (2016) No fixed item limit in visuospatial working memory. Cortex 83:181–193. 10.1016/j.cortex.2016.07.021 27565636 PMC5043407

[B54] Sestieri C, Shulman GL, Corbetta M (2017) The contribution of the human posterior parietal cortex to episodic memory. Nat Rev Neurosci 18:183–192. 10.1038/nrn.2017.6 28209980 PMC5682023

[B55] Stagg CJ, Nitsche MA (2011) Physiological basis of transcranial direct current stimulation. Neuroscientist 17:37–53. 10.1177/1073858410386614 21343407

[B56] Teng C, Postle BR (2024) Investigating the roles of the visual and parietal cortex in representing content versus context in visual working memory. eNeuro 11:ENEURO.0270-20.2024. 10.1523/ENEURO.0270-20.2024 38336475 PMC10860598

[B57] Terney D, Chaieb L, Moliadze V, Antal A, Paulus W (2008) Increasing human brain excitability by transcranial high-frequency random noise stimulation. J Neurosci 28:14147–14155. 10.1523/JNEUROSCI.4248-08.2008 19109497 PMC6671476

[B58] Todd JJ, Marois R (2004) Capacity limit of visual short-term memory in human posterior parietal cortex. Nature 428:751–754. 10.1038/nature02466 15085133

[B59] Tseng P, Hsu TY, Chang CF, Tzeng OJ, Hung DL, Muggleton NG, Walsh V, Liang WK, Cheng SK, Juan CH (2012) Unleashing potential: transcranial direct current stimulation over the right posterior parietal cortex improves change detection in low-performing individuals. J Neurosci 32:10554–10561. 10.1523/JNEUROSCI.0362-12.2012 22855805 PMC6621415

[B60] van Lamsweerde A, Johnson J (2015) The role of the occipital cortex in capacity limits and precision of visual working memory. J Vis 15:661. 10.1167/15.12.661

[B61] Vergallito A, Feroldi S, Pisoni A, Romero Lauro LJ (2022) Inter-individual variability in tDCS effects: a narrative review on the contribution of stable, variable, and contextual factors. Brain Sci 12:522. 10.3390/brainsci12050522 35624908 PMC9139102

[B62] Vogel EK, Machizawa MG (2004) Neural activity predicts individual differences in visual working memory capacity. Nature 428:748–751. 10.1038/nature02447 15085132

[B63] Wagner AD, Shannon BJ, Kahn I, Buckner RL (2005) Parietal lobe contributions to episodic memory retrieval. Trends Cogn Sci 9:445–453. 10.1016/j.tics.2005.07.001 16054861

[B64] Wang S, Itthipuripat S, Ku Y (2019) Electrical stimulation over human posterior parietal cortex selectively enhances the capacity of visual short-term memory. J Neurosci 39:528–536. 10.1523/JNEUROSCI.1959-18.2018 30459222 PMC6335754

[B65] Wang S, Itthipuripat S, Ku Y (2020) Encoding strategy mediates the effect of electrical stimulation over posterior parietal cortex on visual short-term memory. Cortex 128:203–217. 10.1016/j.cortex.2020.03.005 32361592

[B66] Wischnewski M, Berger TA, Opitz A, Alekseichuk I (2024) Causal functional maps of brain rhythms in working memory. Proc Natl Acad Sci U S A 121:e2318528121. 10.1073/pnas.2318528121 38536752 PMC10998564

[B67] Xiao X, Dong Q, Gao J, Men W, Poldrack RA, Xue G (2017) Transformed neural pattern reinstatement during episodic memory retrieval. J Neurosci 37:2986–2998. 10.1523/JNEUROSCI.2324-16.2017 28202612 PMC6596730

[B68] Xie Y, Xu Y, Bian C, Li M (2017) Semantic congruent audiovisual integration during the encoding stage of working memory: an ERP and sLORETA study. Sci Rep 7:5112. 10.1038/s41598-017-05471-1 28698594 PMC5505990

[B69] Zhang W, Luck SJ (2008) Discrete fixed-resolution representations in visual working memory. Nature 453:233–235. 10.1038/nature06860 18385672 PMC2588137

[B70] Zhang Y, Zhang Y, Cai P, Luo H, Fang F (2019) The causal role of alpha-oscillations in feature binding. Proc Natl Acad Sci U S A 116:17023–17028. 10.1073/pnas.1904160116 31383766 PMC6708338

[B71] Zokaei N, Heider M, Husain M (2014) Attention is required for maintenance of feature binding in visual working memory. Q J Exp Psychol 67:1191–1213. 10.1080/17470218.2013.852232 24266343 PMC4047630

