# Erratum: Ye et al., “Transcranial Direct Current Stimulation over the Posterior Parietal Cortex Increases Nontarget Retrieval during Visual Working Memory”

**DOI:** 10.1523/ENEURO.0569-24.2024

**Published:** 2025-01-17

In the article “Transcranial Direct Current Stimulation Over the Posterior Parietal Cortex Increases Nontarget Retrieval During Visual Working Memory,” by Shengfeng Ye, Menglin Wu, Congyun Yao, Gui Xue, and Ying Cai, which published online on November 5, 2024, the Ministry of Education of Humanities and Social Science Project grant number appeared incorrectly. The grant number should read 21YJC190002. The authors apologize for this error, and the online version has been corrected. The full acknowledgment statement appears below.

This work was supported by the National Natural Science Foundation of China (32100851), the Ministry of Education of Humanities and Social Science Project (21YJC190002), the Zhejiang Provincial Natural Foundation Grant (LQ21C090005), STI 2030-Major Projects (2021ZD0200409), and the Open Research Fund of the State Key Laboratory of Cognitive Neuroscience and Learning (CNLYB1903).

